# Heat Acclimation with Blood Flow Restriction Improves Cognitive‐Motor Dual‐Task Ability and Neuromuscular Fatigue

**DOI:** 10.1111/sms.70282

**Published:** 2026-04-30

**Authors:** Thomas Goepp, Oliver R. Gibson, Thomas Rupp, Mark Hayes

**Affiliations:** ^1^ Inter‐University Laboratory of Human Movement Sciences, LIBM University Savoie Mont‐Blanc Chambéry France; ^2^ Department of Sport, Health and Exercise Sciences Brunel University of London Uxbridge UK; ^3^ Center for Magnetic Resonance in Biology and Medicine, UMR CNRS 6612 Faculty of Medicine of Marseille Marseille France; ^4^ Environmental Extremes Laboratory, School of Education, Sport and Health Sciences University of Brighton Brighton UK

**Keywords:** attention, femoral stimulation, heat acclimation, self‐paced cycling, temperature

## Abstract

This study examined whether heat acclimation (HA), and HA with blood flow restriction (BFR) could attenuate cognition and neuromuscular function impairments in cognitive‐motor dual‐task (CMDT) during exercise‐heat stress. Twenty trained adults were randomly assigned to one of two HA protocols over six sessions (~8 days). Each session consisted of 4 × 8‐min self‐regulated cycling intervals (5‐min “strong effort”, 3‐min “moderate effort”) in temperate conditions (20°C), immediately followed by 40‐min hot water immersion (40°C). One group exercised with BFR during the “strong” bouts (50% arterial occlusion pressure; BFR_HA_), whereas the control group completed without occlusion (CTRL_HA_). Before and after HA protocols, participants completed an exercise‐heat stress test with CMDT (pre‐ post‐HA; 40°C, 40% relative humidity): 1 × 7‐min self‐regulated cycling (“strong effort”) followed by 2 × 7‐min fixed‐power blocks with an attentional task. Heart rate, rectal temperature, and mental effort were recorded, while neuromuscular function (peripheral, central responses) was assessed post‐CMDT during a 1‐min sustained maximal contraction. During HA sessions, heart rate and rectal temperature were comparable between groups (*p* ≥ 0.19), despite a reduced power during BFR_HA_ (−22% ± 6%; *p* < 0.001). Post‐HA, both groups reduced (*p* ≤ 0.003) peak heart rate (CTRL_HA_:−5 ± 4 bpm, BFR_HA_:−7 ± 6 bpm) and rectal temperature (CTRL_HA_:−0.19 ± 0.15°C, BFR_HA_:−0.15 ± 0.12°C). Both improved attentional performance (CTRL_HA_:+10% ± 5%, BFR_HA_:+9% ± 7%; *p* < 0.001) with lower mental effort, while reduced force loss was evident only in BFR_HA_ post‐HA (+18% ± 12%, *p* = 0.001), consistent with lower central fatigue (*p* = 0.028) versus pre‐HA. Effective heat adaptations were induced by both HA protocols, despite the lower workload with BFR. Both approaches enhanced sustained attention during an exercise‐heat stress with CMDT, while BFR additionally mitigated central fatigue.

## 1 Introduction

Athletes and military personnel are often required to perform under hot and humid conditions, which impair both physical and cognitive performance [1]. These populations are routinely required to manage physical and cognitive demands concurrently, known as cognitive‐motor dual‐task (CMDT) performance [2]. Even in temperate conditions, CMDT impairs cognitive performance and elicits premature central fatigue compared to physical exercise alone, preferentially under high intensities [3, 4]. Recent findings have reported that challenging environments further impair CMDT performance under hypoxic [3] and heat [5] stress compared to control conditions. Although high‐intensity intervals with cognitive training may alleviate these negative effects in temperate environments [6], it remains unknown whether heat acclimation (HA) protocols attenuate the deleterious effects of heat stress on CMDT.

Consensus recommendations highlight that HA is the preferred intervention to mitigate the adverse effects of heat on physical performance [7]. Attenuation of the adverse effects of heat stress is achieved via post intervention reductions in heart rate (HR) and rectal temperature (T_rec_) change/maximality (i.e., internal strain), for a given power output, while reducing central fatigue [8, 9]. However, evidence regarding the effects of HA on cognitive performance under heat stress remains inconsistent, largely due to methodological confounders often associated with cognitive assessments (e.g., single‐ vs. dual‐task, cognitive domain, level of difficulty) [10]. Hypothetically, HA may improve cognitive performance in the heat through reduced internal strain relative to the pre‐HA state, thereby preserving brain functions that can be altered under hyperthermia [11].

Several effective HA interventions exist, all of which share the requirement of maintaining high and sustained internal strain [12]. Among these, post‐exercise hot water immersion (HWI) is an effective practical strategy that induces adaptations in HR and T_rec_ at rest and during exercise in the heat, requiring only a HWI at ⁓40°C following exercise in a temperate environment [13, 14, 15]. However, implementation of this method is traditionally based on constant‐load running calibrated to VO_2_max, which limits its applicability for individuals who lack access to maximal testing or those who must minimize external mechanical loads, such as the significant joint stress associated with running [16].

Exercising with blood flow restriction (BFR), an intervention to optimize the ratio between internal and external load, is particularly beneficial for athletes with high training volumes, individuals returning from injury, or those seeking to reduce external load and fatigue during tapering before an important event (e.g., competition or military deployment) [17]. Briefly, BFR involves partially occluding the limbs to reduce oxygen delivery and amplify metabolic stress during exercise, despite the use of minimal external load, applicable to both endurance and resistance training. Subsequently, exercising with BFR would induce greater nociceptive and metabolic perturbations, thereby enhancing group III/IV afferent feedback [18, 19], which may contribute to explain the higher central fatigue observed [20], alongside increased motor units' recruitment [21]. Although no differences may exist in neuromuscular adaptations following BFR‐based or classical training programs (e.g., comparable increase in maximal strength or muscular hypertrophy) [22], it remains unknown how BFR‐based training programs modulate central fatigue induced by a high‐intensity exercise (e.g., overreaching of the motor drive leading to mitigated central fatigue?).

Through this construct, exploring the post‐temperate exercise HWI, combined with BFR, to reduce external load during exercise is relevant and has not been examined conclusively. Whilst independently, HA and BFR‐based training programs have been shown to improve individual fitness level through improved VO_2_max or thermoregulatory efficiency [23, 24], the synergistic effects of combining HA and BFR have not been examined. Further to this, whether independent and combined HA and BFR interventions can attenuate the decline in central fatigue and CMDT performance in the heat remains unknown. One may argue that, due to the reduced external load induced by BFR, metabolic heat production and overall internal strain related to heat storage may be attenuated, despite a higher level of muscular acidosis [25]. Consequently, the rise in T_rec_ may be blunted, potentially leading to a sub‐optimal heat stimulus for inducing heat adaptations during HA sessions integrating BFR.

The present study therefore aimed to investigate the effects of a HA protocol combining temperate exercise with BFR prior to HWI, on (i) markers of HA (e.g., T_rec_, HR, sweat rate, plasma volume, thermal comfort), (ii) CMDT performance in the heat (i.e., sustained attention superimposed to high‐intensity cycling exercise), and (iii) neuromuscular fatigue in comparison to HA via post‐exercise HWI alone. We hypothesized that post‐exercise HWI without BFR would elicit greater improvements in thermoregulatory responses (owing to higher internal strain), leading to enhanced CMDT performance during cycling in the heat than HA combined with BFR. Conversely, we further hypothesized that neuromuscular fatigue would be most greatly attenuated following the combined HA and BFR protocol.

## 2 Materials and Methods

### 2.1 Participants

Twenty healthy trained adults were randomly allocated to one of two HA groups: temperate condition cycling prior to HWI (CTLR_HA_: *n* = 10, age: 34 ± 12 years, body mass: 78 ± 11 kg, body mass index: 24.2 ± 2.0 kg·m^−2^, training per week: 9 ± 3 h) or temperate condition cycling with BFR prior to HWI (BFR_HA_: *n* = 10, age: 35 ± 12 years, body mass: 74 ± 14 kg, body mass index: 23.1 ± 3.8 kg·m^−2^, training per week: 8 ± 2 h). All participants were Tier 2 according to the participant classification framework [26] and the main sports identified were triathlon, cycling and running. Due to resource constraints (i.e., absence of similar experimental design) and specific methodological considerations [27], we limited our sample size to 20 participants. This choice allowed us to mitigate the potential interference of natural HA, which can be driven by fluctuating ambient temperatures. Consequently, the laboratory‐based HA protocol was conducted within a narrow seasonal window (February and March). Participants refrained from intense or unhabitual physical exercise within the 24 h before visit, avoided any consumption of analgesics, alcohol, caffeine, or energetic beverages on visit days and slept for at least 7 h the night before the visits. The study followed the principles of the Declaration of Helsinki (2008 edition) and was approved by the institutional ethics committee (2024–14 446). All participants provided written informed consent prior to the study and were naive to the study aims and hypotheses.

### 2.2 Experimental Design

Participants visited the laboratory for a total of ten visits, including two preliminary visits (i.e., incremental cycling test and familiarization), six HA sessions completed over ⁓1 week and two testing visits for pre‐ and post‐HA sessions. Participants were randomly assigned to one of the two HA groups (CTLR_HA_ or BFR_HA_) using a stratified randomization procedure to balance age, gender and peak power output (data collection described in *Preliminary visits* section).

A two‐group comparative design was adopted to specifically evaluate the effects of BFR within a validated short‐term HA framework using post‐exercise HWI [13, 14, 15]. Although we utilized high‐intensity intervals instead of constant‐load exercise, primarily for BFR safety and efficacy concerns [28], this framework was chosen to prioritize the study of synergistic adaptations between thermal and metabolic stressors. Furthermore, an exercise‐only control group was not included as six sessions of short‐duration intervals are unlikely to yield significant aerobic training adaptations in highly trained individuals, thus minimizing the risk of a confounding training effect [29].

### 2.3 Preliminary Visits: Incremental Cycling Test and Familiarization Session

*1st visit*. The first visit was designed to provide data to (i) homogenize training groups based on achieved peak power output (i.e., stratified randomization) and (ii) express cycling power and HR as a percentage of peak power output and maximal HR (%PPO and %HR_max_, respectively). Participants therefore performed an incremental cycling test on a stationary cycle ergometer (Wattbike Ltd., Nottingham, UK) with a cardiothoracic girdle sensor (Polar E10, Kempele, Finland) to collect peak power output (323 ± 60 W) and HR_max_ (180 ± 13 bpm). The first stage started at 140 W, with work‐rate increased by 15 W every minute until volitional exhaustion, despite strong verbal encouragement. Peak power output was determined as the last 1‐min completed stage.

*2nd visit*. The second visit was conducted to (i) familiarize the participants with cycling at various levels of perceived effort (i.e., self‐regulated power), using the 100‐points Borg scale (CR_100_) [30], (ii) determine preferential cadence, and (iii) introduce procedures for neuromuscular function assessment. The participants also completed the sustained attention to response time task (SART, see *Cognitive task* section) at rest (1 × 60 trials) and while cycling at a self‐regulated power corresponding to a perceived effort of 50 a.u. (arbitrary units), defined as “strong” on the CR_100_ (1 × 120 trials). At the end of the familiarization, arterial occlusion pressure was assessed by inflating the 8‐cm wide BFR cuff (The Occlusion Cuff Ltd., Belfast, Ireland) placed around the most proximal portion (inguinal fold) of the right thigh while the participant lay supine. Pressure was gradually increased until the Doppler probe (Elica, Hambourg, Germany) failed to detect a pulse (popliteal artery pulse in popliteal fossa) distal to the cuff, confirming full arterial occlusion (242 ± 37 mmHg).

### 2.4 Pre‐ and Post‐HA Visits: Heat Stress Test With CMDT (HST_CMDT_ )

**FIGURE 1:**
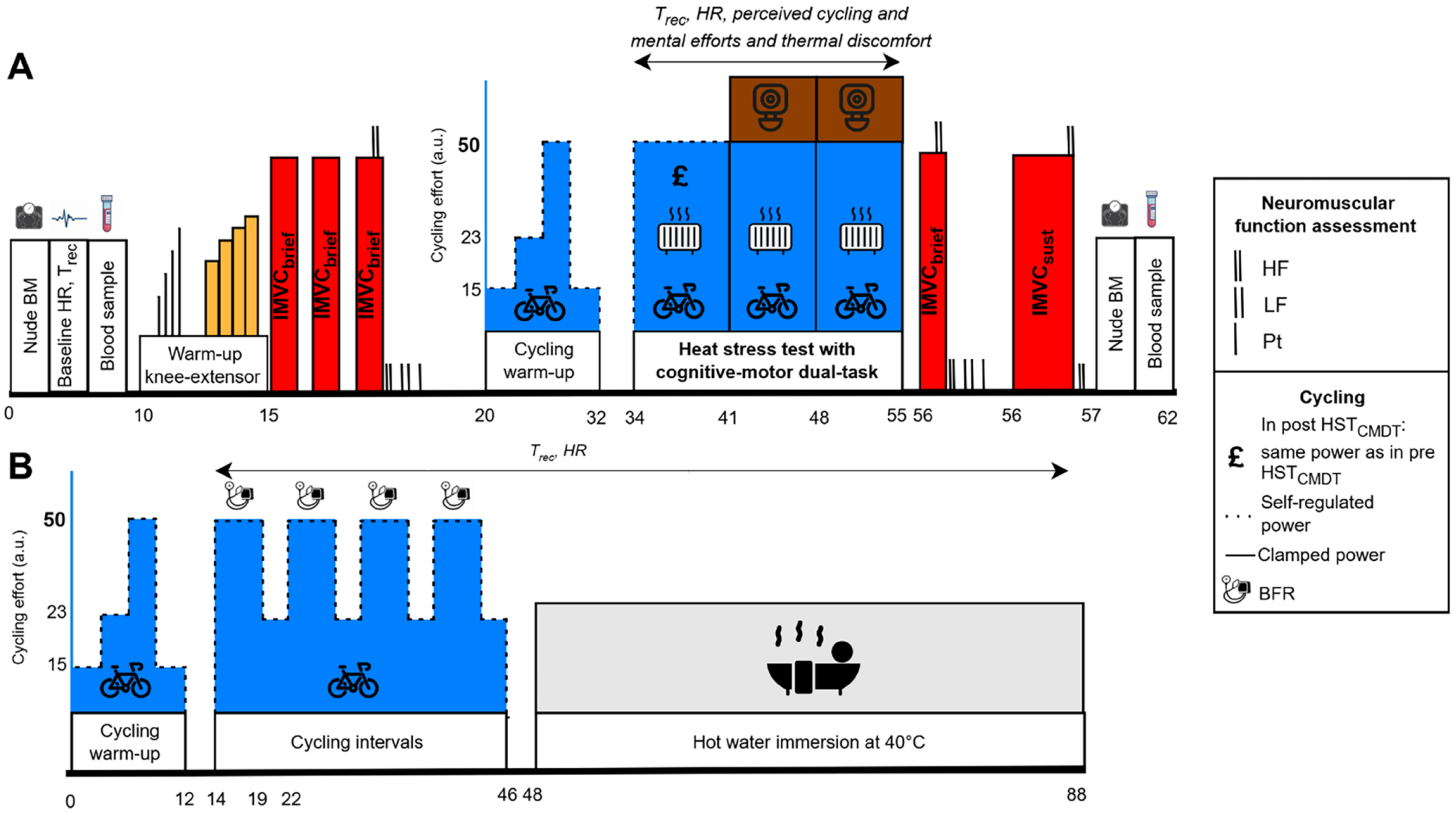
Overview of the experimental procedures: Pre and post heat stress test with cognitive‐motor dual‐task (HST_CMDT_, panel A) visits, and schematic representation of a HA session including post‐cycling hot water immersion (panel B). BM, Body mass; BFR, Blood flow restriction; HF, High‐frequency doublet; HR, Heart rate; IMVC_brief_, 3‐s brief isometric maximal voluntary contraction; IMVC_sust_, 1‐min sustained isometric maximal voluntary contraction; LF, Low‐frequency doublet; Pt, Peak twitch; T_rec_, Rectal temperature.

*3rd and 10th visits*. Upon arrival, nude body mass was measured (Adam GFK 150 Body Scales, Connecticut, USA) and resting HR and T_rec_ were recorded after 5 min seated rest in a temperate environment (~20°C). A fingertip‐blood sample was collected to determine hematocrit and neuromuscular function of the right knee‐extensors was assessed. The participants then completed a 12‐min progressive cycling warm‐up (i.e., 4 × 3 min at 13, 23, 50 and 23 a.u. on the CR_100_, respectively corresponding to “easy”, “moderate”, “strong” and “moderate” cycling efforts). Then, the participants entered the environmental chamber (TISS, Hampshire, UK) which was set at 40.0°C ± 0.2°C, 40.2% ± 0.5% relative humidity. They completed 21 min of cycling exercise divided into 3 × 7‐min blocks: 1 × 7 min of self‐regulated cycling (Ex0‐7) at a power corresponding to a perceived effort of 50 a.u., defined as “strong” on the CR_100_ and then, 2 × 7 min at a clamped cycling power based on the final power selected by the participant during Ex0‐7 (Ex7‐14, Ex14‐21). The participants were asked to maintain their preferential cadence throughout the cycling task (85 ± 5 RPM), with visual feedback available. Importantly, the participants performed the SART task during Ex7‐14 and Ex14‐21 (cf. CMDT). Upon completion of the 21‐min cycling in the heat, participants exited the chamber and completed neuromuscular function assessment including a brief isometric maximal voluntary contraction (IMVC) followed by a sustained IMVC (see *Neuromuscular function assessment* section). This procedure limited misinterpretation of voluntary activation (VA) reduction due to the delay between exiting the chamber and initiating the right knee‐extensors contraction (i.e., ⁓30 s) [31]. A second fingertip‐blood sample was analyzed for hematocrit and dry nude body mass was then obtained before resting for 15 min, during which time cooling was applied (e.g., cold water consumption, fanning and immersion of feet in cold water) to aid recovery. No fluid was consumed between both nude body mass measurements. The post‐HA visit (post HST_CMDT_) was scheduled 48 to 72 h after the last HA session and cycling power was set based on the last power chosen during Ex0‐7 from the pre HST_CMDT_ visit, facilitating iso‐power comparisons. An overview of the assessment visits is presented in Figure 1A.

*Cognitive task*. Sustained attention was engaged using the SART task during the last two 7‐min blocks of cycling in the heat, using the web‐based resource PsyToolkit [32]. The SART involved numbers from 1 to 9 randomly displayed on a computer screen for 250 ms each, followed by an 800‐ms response window with participants instructed to press a button (fixed on the cycle ergometer) as quickly as possible for all numbers, except for the number 3, for which they had to withhold their response (11% of all trials). Therefore, participants needed to be both as fast and as accurate as possible. SART score, which equally weighed reaction time and accuracy, was evaluated and averaged within each 7‐min block. Prior to data collection, participants were provided with a comprehensive description of the task and its instructions. At the beginning of the pre and post HST_CMDT_ visits, participants completed a brief practice block (30 trials, ~30 s) as a reminder of the task mechanics. The SART was demonstrated as reliable (ICC_(3,1)_ = 0.78) and without learning effect (*p* > 0.05) during a subgroup analysis (see Data S1 for details).

*Cycling ergometer*. Cycling trials in the heat were conducted on a stationary cycle ergometer (Wattbike Ltd., Nottingham, UK) with self‐regulated airflow resistance, adjusted by participants via a manually operated flap mechanism. The coefficient of variation between two repeated functional threshold power tests performed on a Wattbike has previously been reported as 2.6% [33]. Power output was averaged and presented over the clamped cycling phases (CMDT, Ex7‐21). As a reminder, during the post HST_CMDT_ visit, participants cycled at the same power output they had self‐selected during the pre HST_CMDT_ visit.

*Thermoregulatory variables: HR, T*_*rec*_, *sweat rate and change in plasma volume*. HR was recorded using a cardiothoracic girdle sensor. HR data were averaged over the 5‐min resting state in a temperate environment, and over the first and last 30 s of cycling to estimate the HR change within cycling [HR_end_—HR_start_]. T_rec_ was monitored using a single rectal temperature probe (449H, Henleys Medical, Hertfordshire, UK), self‐inserted 10 cm past the anal sphincter, and displayed on logging monitors (YSI, 4600 series, YSI, Hampshire, UK). T_rec_ was collected as single values at the end of the 5‐min resting state, and during the start and end of cycling to estimate the T_rec_ change within cycling [T_rec(end)_ – T_rec(start)_]. To provide an objective internal intensity estimation of the HST_CMDT_, peak HR and T_rec_ are presented in Table 1.

**TABLE 1:** Clamped cycling power output during Ex7‐21 expressed as absolute and percentage of peak power output (%PPO) and thermoregulatory variables including heart rate (HR), rectal temperature (T_rec_), sweat rate and plasma volume change in pre and post heat stress test with cognitive‐motor dual‐task (HST_CMDT_) visits, in the control group (CTRL_HA_), and in the group with blood flow restriction during high‐intensity intervals (BFR_HA_).

	CTRL_HA_		BFR_HA_		*Two‐way ANOVA p*		
	Pre HST_CMDT_	Post HST_CMDT_	Pre HST_CMDT_	Post HST_CMDT_	*HA*	*Group*	*HA × Group*
Clamped Cycling power output (W and %PPO)	199 ± 46 W	200 ± 45	186 ± 45	184 ± 45	0.62	0.52	0.73
	62 ± 6%	62 ± 6	58 ± 6	58 ± 6			
Resting HR (bpm)	58 ± 6	54 ± 8	59 ± 8	56 ± 7	**0.014** (0.29)	0.55	0.82
Peak HR (bpm and %HR_max_)	177 ± 16 bpm	172 ± 17	170 ± 15	165 ± 13	**0.003** (0.41)	0.30	0.97
	97 ± 4%	94 ± 5	96 ± 4	93 ± 4			
HR change within cycling (𝛥bpm)	28 ± 5	21 ± 8	26 ± 6	20 ± 5	**0.018** (0.24)	0.95	0.77
Resting T_rec_ (°C)	37.22 ± 0.50	37.07 ± 0.39	37.21 ± 0.29	37.22 ± 0.35	0.42	0.64	0.34
Peak T_rec_ (°C)	38.43 ± 0.40	38.25 ± 0.37	38.39 ± 0.37	38.24 ± 0.39	**0.002** (0.43)	0.88	0.76
T_rec_ change within cycling (𝛥°C)	0.89 ± 0.34	0.81 ± 0.3	0.81 ± 0.19	0.69 ± 0.14	**0.047** (0.20)	0.34	0.62
Sweat rate (L·h^−1^)	1.4 ± 0.4	1.8 ± 0.6	1.4 ± 0.4	1.9 ± 0.7	**< 0.001** (0.63)	0.83	0.24
Change in plasma volume (%)	−4.5 ± 3.3	−2.4 ± 3.0	−4.3 ± 3.2	−1.9 ± 3.8	**0.002** (0.43)	0.79	0.80

*Note:* Significant *p*‐values are shown in bold and reported as *p*‐value and (partial eta‐squared).

Changes in body mass were used to estimate whole body sweat rate as:

$$\text{Sweat rate}\;\left(L\cdot h^{-1}\right)=\left(\frac{\left({\mathrm{BM}}_{\text{before}}-{\mathrm{BM}}_{\text{after}}\right)}{\text{Exercise time}}\;\right)\times 60$$

where BM_before_ and BM_after_ are respectively dry nude body masses before and after the 21‐min cycling in the heat.

To calculate change in plasma volume we measured hematocrit. Fingertip capillary blood samples were collected and centrifuged (Hawksley haematospin 1300, Lancing, England) at 1300 RPM for 2 min. Hematocrit was measured using a micro hematocrit reader (Hawksley, Lancing, England), allowing calculation of plasma volume before and after the 21‐min cycling in the heat [34]:

$$\text{Plasma volume}\;\left(L\right)=\mathrm{BV}\times \left(1-\text{Haematocrit}\right)$$

where blood volume (BV) is estimated through the height and bodyweight formula [35]. Plasma volume change was then calculated [36] as:

$$\text{Plasma volume change}\;\left(\%\right)=\left(\frac{\left({\mathrm{PV}}_{\text{after}}-{\mathrm{PV}}_{\text{before}}\right)}{{\mathrm{PV}}_{\text{before}}}\;\right)\times 100$$

where PV_before_ and PV_after_ are respectively the plasma volume calculated before and after the HST_CMDT_.

*Hydration assessment*. Before beginning the HST_CMDT_ visits, participants provided a urine sample to verify euhydration (urine osmolality ≤ 700 mOsm·kg^−1^ H_2_O and specific gravity ≤ 1.020). Four participants in pre HST_CMDT_ and five at post HST_CMDT_ visits did not meet these criteria; therefore, 500 mL of water was provided. No participants reached the heat‐termination criteria (i.e., T_rec_ ≥ 39.7°C or an increase of ≥ 2.0°C from resting T_rec_ or if participants requested).

*Neuromuscular function assessment*. To assess peripheral and central indices of neuromuscular fatigue, electrical stimulations were administered percutaneously to the femoral nerve via a cathode electrode (10‐mm diameter; Meditrace 100, Covidien) placed on the inguinal triangle. A rectangular anode electrode (50 × 90‐mm, Durastick Plus, DJO Global, Vista, CA, USA) was attached to the gluteal fold. An electrically‐induced square wave of 1‐ms duration was delivered using a constant current stimulator (DS7A, Digitimer, Welwyn Garden City, Hertfordshire, UK). Before starting the procedure, the optimal intensity of stimulation was determined using single stimuli, delivered incrementally in steps of 10 mA, every 5 s, until the twitch force amplitudes plateaued. The optimal intensity was then increased by 20% for subsequent evaluations to ensure supramaximality. Then, a warm‐up was completed and included progressively increased isometric contractions of the right knee‐extensors.

Brief IMVC assessments (IMVC_brief_) were performed before (baseline) and 45 s post‐CMDT (i.e., standardized delay to transit the participant from the environmental chamber to the knee‐extensors ergometer). This evaluation was composed of a 3‐s IMVC with one high‐frequency doublet at 100 Hz evoked during the force plateau. Starting 3‐s after the IMVC_brief_, three electrical stimuli were delivered to the relaxed muscle interspersed with 3 s between each stimulus: one high‐frequency doublet, one low‐frequency doublet at 10 Hz, and one peak twitch. IMVC_brief_ and peak twitch amplitude changes within the session (from baseline to post‐CMDT), low‐frequency to high‐frequency ratio (i.e., low‐frequency fatigue index) and VA (see formula below) were calculated [37, 38].

Following a 5‐s transition after the last twitch, the participants realized a 1‐min sustained IMVC (IMVC_sust_). A superimposed high‐frequency doublet was applied at 55 s during the IMVC_sust_ and again 3 s after relaxation. IMVC_sust_ change within the contraction (from the start to the end of the task) and VA at +55 s were calculated.

$$\mathrm{VA}\left(\%\right)=\left(\;1-\frac{\mathrm{SIT}*\frac{F_{\text{init}}}{\text{IMVC}}}{{\mathrm{HF}}_{\text{relaxed muscle}}}\;\right)*100$$

where SIT is the amplitude evoked when the high‐frequency doublet (HF) was delivered on IMVC_brief_ and IMVC_sust_, F_init_ is the force right before the stimulation.

*Perceived cycling and mental efforts and thermal discomfort*. Cycling effort, asked as “What is the difficulty of your cycling exercise?” [39] and mental effort asked as “What is the mental effort associated with the situation?” [40] were assessed using the CR_100_. Thermal discomfort was assessed using the question “How hot do you feel right now?” rated on a 10‐cm visual analogue scale ranging from 0 (“neutral”) to 10 (“unbearably hot”). All measurements were assessed at the end of each 7‐min cycling block. As little evidence exists considering BFR‐based training programs and pain tolerance [22], we also assessed perceived quadriceps muscle pain during the HST_CMDT_ with the following question: “Rate the intensity of pain you feel specifically in your quadriceps muscles right now”, using the CR_100_. All questions were administered during the final 30 s of each 7‐min block (Ex7, Ex14, and Ex21) during both pre and post HST_CMDT_ visits.

### 2.5 HA Sessions (⁓1 Week)

*4th to 9th visits*. The HA protocol followed a short‐term HA framework using post‐exercise HWI, as previously described and validated [15]. Each group completed six HA sessions, theoretically scheduled on consecutive days but actually performed over 8 ± 1 days due to participants' personal and professional constraints (Figure 1B). Each HA session started with a 12‐min warm‐up at progressively increasing cycling intensities on the same cycle used during the pre and post HST_CMDT_ visits, followed by 4 × 8‐min self‐regulated cycling intervals (5‐min at 50 a.u. “strong effort”, 3‐min at 23 a.u. “moderate effort”), using a preferential cadence, in a temperate environment (20°C, 40% relative humidity). During the 5‐min at 50 a.u. “strong effort” intervals, the BFR cuffs were inflated at 5% arterial occlusion pressure for the CTRL_HA_ group or 50% for the BFR_HA_ group around the most proximal portion (inguinal fold) of both thighs and deflated during the 3‐min at 23 a.u. “moderate effort” intervals. An intermittent modality was chosen to maximize BFR cuff application, in accordance with safety guidelines [28]. At the cessation of cycling intervals, dressed in shorts, participants submerged to the neck in a bath filled with water maintained at a temperature of 40.1°C ± 0.5°C for 40 min. Participants were allowed to drink temperate water ad libitum.

*Training load metrics*. The metric retained for analysis was the average self‐regulated cycling power across the 4 sets of 5‐min “strong” intervals. T_rec_ and HR were considered at three time points: rest, end of cycling, and end of HWI. Data were recorded for the first (S1) and last (S6) HA sessions, with detailed session results provided in Data S2.

*Hydration assessment*. The same urine assessments as during the HST_CMDT_ visits were performed, with 6 ± 2 participants not meeting the euhydration criteria at the beginning of each HA session. Therefore, 500 mL of water was provided.

### 2.6 Statistical Analysis

All statistical analyses were performed using JASP software (version 19, Amsterdam, The Netherlands) and statistical significance was set at *p* < 0.05. Normality of residuals was assessed using Shapiro–Wilk tests. Homogeneity of variances, independence of observations, and the presence of extreme outliers were evaluated where applicable, primarily through visual inspection of residual plots, and no significant violations were observed. When sphericity was violated (Mauchly's test), Greenhouse Geisser corrections were applied.

*Pre and post HST*_*CMDT*_ *visits*. To examine the effects of the two HA groups on the SART score (i.e., CMDT) and perceptual responses in the heat, three‐way repeated‐measures analysis of variance (ANOVA) with main effect of *HA* (pre HST_CMDT_, post HST_CMDT_) *× group* (CTLR_HA_, BFR_HA_) *× time intra* (SART score: Ex7‐14 and Ex14‐21, perceptual responses: Ex7, Ex14 and Ex21) was performed. For thermoregulatory and neuromuscular indices two‐way repeated‐measures ANOVAs with main effect of *HA* (pre HST_CMDT_, post HST_CMDT_) *× group* (CTLR_HA_, BFR_HA_) were conducted. To capture and account for interindividual differences, particularly at the points corresponding to Ex14‐21 (SART score) or Ex21 (perceptual responses, HR and T_rec_ peak), complementary analysis was conducted using two‐way repeated‐measures ANOVAs with main effect of *HA* (pre HST_CMDT_, post HST_CMDT_) *× group* (CTLR_HA_, BFR_HA_).

*HA sessions*. To examine the effects of the two HA groups on self‐regulated cycling power, HR and T_rec_ indices (rest, cycling end and HWI end) two‐way repeated‐measures ANOVAs with main effect of *HA* (S1, S6) *× group* (CTLR_HA_, BFR_HA_) were conducted.

Pairwise Bonferroni procedures were applied for post hoc analyses when ANOVA revealed significant results. The partial eta squared (η_p_^2^) for ANOVA analysis was calculated, where η_p_^2^ < 0.01 indicates a very small effect, 0.01 ≤ η_p_^2^ < 0.06 a small effect, 0.06 ≤ η_p_^2^ < 0.14 a moderate effect, and η_p_^2^ ≥ 0.14 a large effect [41]. To facilitate clarity in the presentation of results, only the main effects of *HA* and *group*, as well as the *HA* × *group* interaction, were reported from the three‐way ANOVAs.

Based on data indicating a positive effect for both HA groups on SART score during cycling in the heat, correlational analysis was performed from pre to post HST_CMDT_. Sustained attention performance (∆SART score) was tested with the level of hyperthermia reached (∆T_rec_) and the level of central fatigue induced (∆VA), as both may contribute to CMDT performance [42, 43].

## 3 Results

### 3.1 Pre and Post HST_CMDT_ Visits

*Thermoregulatory variables*. For resting HR, and HR during exercise (i.e., change within cycling and peak), main effects of *HA* were observed with reduced values in post HST_CMDT_ (all *p* ≤ 0.018), with no *group* effect or *HA × group* interaction (all *p* ≥ 0.30). No *HA* effect, *group* effect or *HA × group* interaction was reported for resting T_rec_ (all *p* ≥ 0.34). For T_rec_ during exercise (i.e., T_rec_ change within cycling and peak T_rec_), main effects of *HA* were observed with reduced values in post HST_CMDT_ (all *p* ≤ 0.047), and no *group* effect or *HA × group* interaction (all *p* ≥ 0.34). For sweat rate, a main effect of *HA* was observed with higher rate in post HST_CMDT_ (*p* < 0.001), and no *group* effect or *HA × group* interaction (all *p* ≥ 0.24). A main effect of *HA* was observed for plasma volume change throughout the test (*p* = 0.002), with reduced losses in post HST_CMDT_ visits. No *group* effect or *HA × group* interaction was reported for plasma volume changes (all *p* ≥ 0.79). Further data and statistical outcomes relating to thermoregulatory variables are provided in Table 1.

**FIGURE 2:**
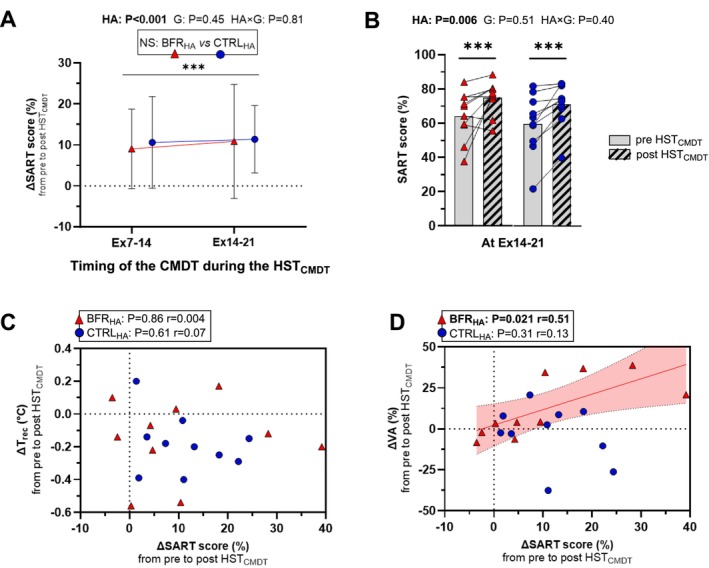
Sustained attention to response time (SART) score from pre HST_CMDT_ visit to post HST_CMDT_ visit (panel A), individual raw data at Ex14‐21 (panel B) and correlations between ΔSART score and Δrectal temperature (T_rec_, panel C) or Δvoluntary activation (VA, panel D) in the control group (CTRL_HA_), and in the group with blood flow restriction during high‐intensity intervals (BFR_HA_). For example, a positive value of ΔSART score at Ex14‐21 in panel A indicates that SART score is increased compared to pre HST_CMDT_ visit at this time‐point. A boxed text presents the *group* effect (panel A) and *p*‐values for main effects of *heat acclimation* (HA), *group* (G) and *heat acclimation × group* interaction (HA *×* G) are displayed above the panels A and B. *p*‐values from the correlation analyses are displayed above panels C and D. The solid line indicates the significant linear regression, and the shaded area represents the 95% confidence interval for the latter. Post hoc statistically significant difference observed for *HA* effect is depicted with the following symbol: ***p < 0.001.

*Cognitive performance*. For SART score, a main effect of *HA* (F_(1,18)_ = 24, *p* < 0.001, η_p_^2^ = 0.58) was reported (Figure 2A,B), with no *group* effect (*p* = 0.45) or *HA × group* interaction (*p* = 0.81). SART score was increased in post HST_CMDT_ (*p* < 0.001) during Ex7‐14 (+10 ± 5%) and Ex14‐21 (+11 ± 5%) in both groups. Correlation analyses revealed that ΔSART score was not correlated with ΔT_rec_ throughout the tests for both groups (all *p* ≥ 0.61, Figure 2C) and positively correlated with ΔVA only in the BFR_HA_ group (*r* = 0.51, *p* = 0.021, Figure 2D).

**FIGURE 3:**
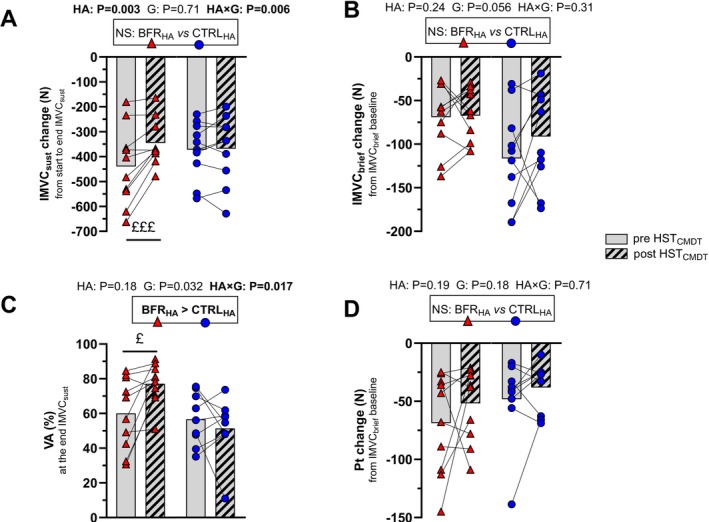
Force loss and voluntary activation (VA) during the sustained isometric maximal voluntary contraction (IMVC_sust_, panels A/C) and force loss and peak twitch (Pt) changes during the brief IMVC (IMVC_brief_, panels B/D) at pre and post HST_CMDT_ in the control group (CTRL_HA_) and in the group with blood flow restriction during high‐intensity intervals (BFR_HA_). As a reminder, the neuromuscular fatigue assessment included post‐CMDT an IMVC_brief_, immediately followed by IMVC_sust_. A boxed text presents the *group* effect and *p*‐values for the main effect of *heat acclimation* (HA), *group* (G), and *heat acclimation × group* interaction (HA *×* G) displayed above the figures. Post hoc statistically significant differences observed for *HA × group* interaction are depicted with the following symbols: ^£^ *p* = 0.048, ^£££^ *p* < 0.001.

*Neuromuscular function*. The change in IMVC_sust_ presented a main effect of *HA* (F_(1,18)_ = 11, *p* = 0.003, η_p_^2^ = 0.39), no *group* effect (*p* = 0.71), and a main *HA × group* interaction (F_(1,18)_ = 10, *p* = 0.006, η_p_^2^ = 0.35) (Figure 3A). The decline in IMVC_sust_ was attenuated in post HST_CMDT_ compared to pre HST_CMDT_, only for BFR_HA_ (pre HST_CMDT_ = −439 ± 157 N, post HST_CMDT_ = −345 ± 93 N; *p* = 0.001). VA collected at the end of IMVC_sust_ presented no *HA* effect (*p* = 0.18), and a main effect of *group* (F_(1,18)_ = 5, *p* = 0.032, η_p_^2^ = 0.24) and *HA × group* interaction (F_(1,18)_ = 7, *p* = 0.017, η_p_^2^ = 0.29) (Figure 3C). VA was better preserved at the end of IMVC_sust_ in post HST_CMDT_ compared to pre HST_CMDT_, only following BFR_HA_ (pre HST_CMDT_ = 60 ± 21%, post HST_CMDT_ = 77 ± 12%; *p* = 0.048). For the change in IMVC_brief_ and peak twitch, no effect of *HA* (*p* = 0.24 and *p* = 0.19, respectively), *group* (*p* = 0.056 and *p* = 0.18), or *HA × group* interaction (*p* = 0.31 and *p* = 0.72) was observed (Figure 3B,D). Data on low‐frequency to high‐frequency ratio and VA from IMVC_brief_ in pre and post HST_CMDT_ visits are presented in Data S3, with no main effect of *HA*, *group*, or *HA × group* interaction.

**FIGURE 4:**
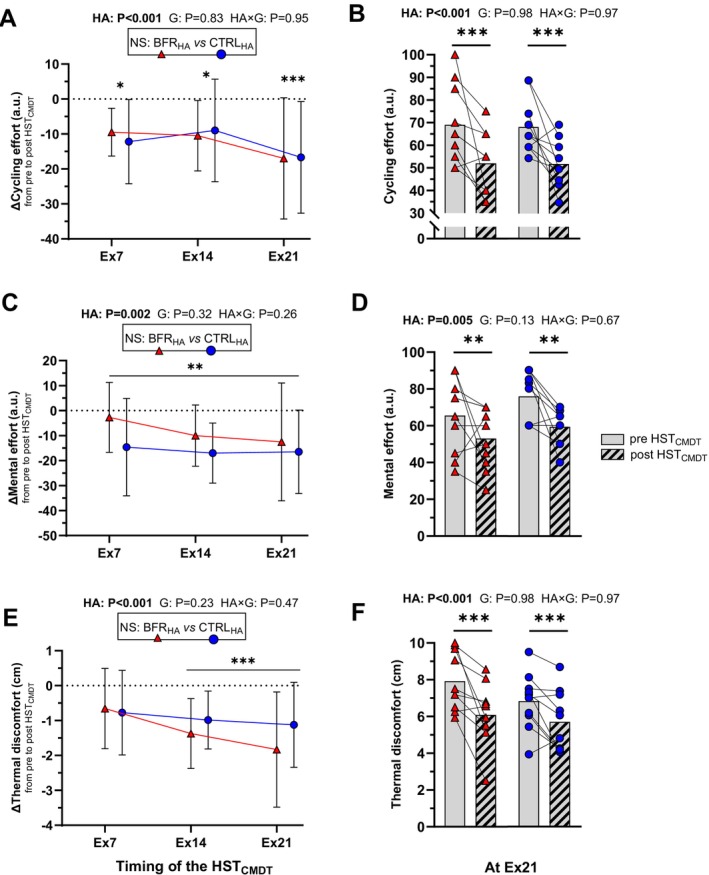
Perceived cycling and mental effort and thermal discomfort from pre HST_CMDT_ visit to post HST_CMDT_ visit (panels A/C/E) and respective individual raw data at Ex21 (panels B/D/F) in the control group (CTRL_HA_), and in the group with blood flow restriction during high‐intensity intervals (BFR_HA_). For example, a negative value of Δmental effort at Ex21 in panel C indicates that perceived mental effort is reduced compared to pre HST_CMDT_ visit at this time‐point. A boxed text presents the *group* effect (panel A) and *p*‐values for main effects of *heat acclimation* (HA), *group* (G) and *heat acclimation × group* interaction (HA *×* G) are displayed above the panels. Post hoc statistically significant differences observed for *HA × time intra* interaction or *HA* effect are depicted with the following symbols: *p ≤ 0.037, **p ≤ 0.005, ***p < 0.001.

*Perceived cycling and mental effort and thermal discomfort*. For cycling effort, a main effect of *HA* (F_(1,18)_ = 25, *p* < 0.001, η_p_^2^ = 0.58) was observed (Figure 4A,B), with no *group* effect (*p* = 0.83) or *HA × group* interaction (*p* = 0.95). Cycling effort was decreased in post HST_CMDT_ (−12 ± 6 a.u., *p* < 0.001) in both groups. For mental effort, a main effect of *HA* (F_(1,18)_ = 14, *p* = 0.002, η_p_^2^ = 0.43) was reported (Figure 4C,D), with no *group* effect (*p* = 0.32) or *HA × group* interaction (*p* = 0.26). Mental effort was decreased in post HST_CMDT_ (−12 ± 6 a.u., *p* = 0.002) in both groups. For thermal discomfort, a main effect of *HA* (F_(1,18)_ = 26, *p* < 0.001, η_p_^2^ = 0.59) was reported (Figure 4E,F), with no *group* effect (*p* = 0.23) or *HA × group* interaction (*p* = 0.47). Thermal discomfort was decreased in post HST_CMDT_ at Ex14 (−12 ± 0.9 mm, *p* = 0.001) and Ex21 (−15 ± 14 mm, *p* < 0.001) in both groups. Data on quadriceps muscle pain in pre and post HST_CMDT_ visits are presented in Data S3.

### 3.2 HA Sessions

**FIGURE 5:**
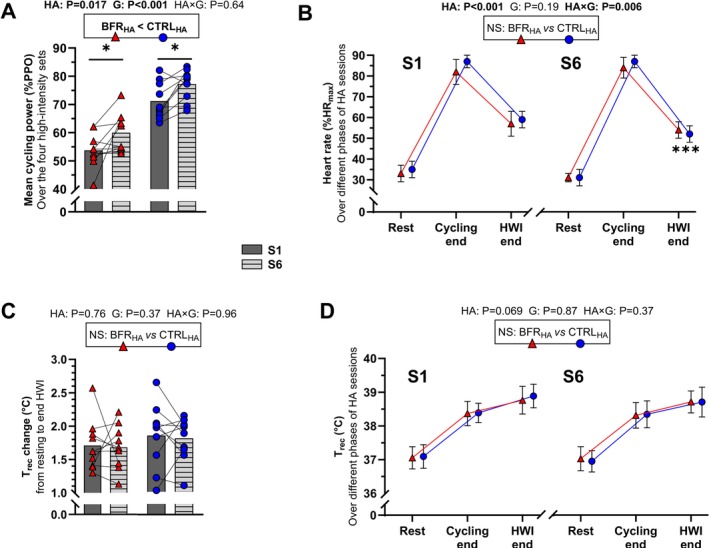
Mean cycling power in % of peak power output (%PPO, panel A), heart rate in % of maximal heart rate (%HR_max_, panel B), change in rectal temperature (T_rec_, panel C), and T_rec_ (panel D) during the first (S1) and last (S6) HA sessions in the control group (CTRL_HA_), and in the group with blood flow restriction during high‐intensity intervals (BFR_HA_). Mean cycling power represents the average power across the four high‐intensity sets (panel A). T_rec_ and HR are presented at rest, at the end of the cycling exercise, and at the end of the hot‐water immersion (HWI, panels B/D). T_rec_ change represents the rise in T_rec_ from rest to the end of HWI as an indicator of internal heat strain (panel C). A boxed text presents the *group* effect and *p*‐values for the main effect of *heat acclimation* (HA), *group* (G), and *heat acclimation × group* interaction (HA *×* G) displayed above the figures. Post hoc statistically significant difference observed for *HA* effect is depicted vs. S1 with the following symbol: *p = 0.017, ***p < 0.001.

*Training load metrics*. For self‐regulated power, a main effect of *HA* (F_(1,18)_ = 7, *p* = 0.017, η_p_^2^ = 0.28) and *group* (F_(1,18)_ = 61, *p* < 0.001, η_p_^2^ = 0.77) was observed (Figure 5A) with no *HA × group* interaction (*p* = 0.64). Self‐regulated power increased between S1 and S6 (+16 ± 10 W, *p* = 0.017) in both groups, but overall reduced self‐regulated power was observed in the BFR_HA_ group (−55 ± 17 W, *p* < 0.001), compared to the CTRL_HA_ group. For T_rec_ measured during the HA sessions (i.e., at rest, the end of the cycling exercise and the end of the HWI), no effect of *HA* (*p* = 0.069), *group* (*p* = 0.87) or *HA × group* interaction (*p* = 0.37) was reported (Figure 5B). The HR presented a main effect of *HA* (F_(1,18)_ = 33, *p* < 0.001, η_p_^2^ = 0.65), no *group* effect (*p* = 0.19) and a main *HA × group* interaction (F_(1,18)_ = 9, *p* = 0.006, η_p_^2^ = 0.35) (Figure 5C). HR was reduced between S1 and S6 in both groups at HWI end (−10 ± 3 bpm, *p* < 0.001).

## 4 Discussion

The present study investigated the effects of intermittent cycling prior to a HWI protocol (CTRL_HA_) compared to cycling with BFR prior to HWI (BFR_HA_) on thermoregulatory variables (e.g., T_rec_, HR, sweat rate), CMDT performance in the heat (i.e., sustained attention superimposed to a cycling exercise), and associated neuromuscular fatigue. The findings partially confirmed our initial hypothesis, revealing three key outcomes: (i) BFR_HA_ induced similar thermoregulatory adaptations to CTRL_HA_ (e.g., −0.17° ± 0.09°C in T_rec_ peak, −5 ± 1 bpm in HR peak, +0.4 ± 0.2 L·h^−1^ in sweat rate) despite consistently reduced cycling power outputs of −22% ± 6% during HA sessions; (ii) both HA protocols improved sustained attention performance by 10% ± 4% during CMDT in the heat; and (iii) only the BFR_HA_ group reduced their force loss during the sustained contraction after the CMDT in the heat by 18% ± 12% following HA, consistent with a lower induction of central fatigue.

### 4.1 Thermoregulatory Responses to Heat Acclimation

The first aim of the study examined whether cycling with BFR prior to HWI, which consistently reduced cycling power (−22% ± 6%) during the intervention, would impair HA due to the lower external load. Our findings did not observe this phenomenon as the improvement in all thermoregulatory variables (i.e., T_rec_, HR, sweat rate, and plasma volume) was similar in both groups with the magnitude of change induced by the interventions comparable to other HA protocols via post‐exercise HWI [23]. Given the magnitude of the physiological adjustments observed across several markers, both groups exhibited changes consistent with the development of a heat‐acclimated phenotype [14, 44]. The equivalent magnitudes of adaptation induced in the CTRL_HA_ and BFR_HA_ groups are likely a reflection of the equivalent potentiating stimuli for heat adaptation achieved across all HA sessions [45]. Participants in the CTRL_HA_ and BFR_HA_ groups experienced comparable thermoregulatory strain during the four high‐intensity cycling bouts of the sessions, with T_rec_ of ~38.3°C and 38.4°C, respectively, and HR of approximately ~85% and 82% of HR_max_. While the BFR_HA_ group performed a lower absolute external workload, the increased metabolic cost and cardiovascular strain associated with BFR likely yielded a comparable level of internal heat production [21, 46]. Consistent with the cycling phase, end‐set T_rec_ and HR during HWI were also comparable between groups (CTRL_HA_: ~38.8°C and 100 bpm; BFR_HA_: ~38.7°C and 99 bpm). Taken together this highlights that BFR maintains a sufficient internal thermal stimulus to trigger adaptations, when applied during intermittent exercise before HWI, at least when exercise perceived intensities are relatively matched (i.e., 50 a.u. “strong effort”). The comparable thermoregulatory stimuli reflect similar internal strains leading to equivalent HA in both groups, as a consistent elevation in T_rec_ is a key potentiating stimulus for heat adaptation [44].

A notable increase (+6% ± 2%) in self‐regulated cycling power was observed between the first and last HA sessions across groups, possibly reflecting participant's improved ability to maintain a sufficient internal strain. This self‐paced approach enables participants to autonomously adjust external load according to their perceived effort, in line with emerging training methods [6]. To our knowledge, this is the first HA protocol to implement a cycling model that combines BFR with perceived‐effort calibration (rather than traditional prescribed intensity thresholds) prior to HWI. This approach may enhance ecological relevance for some cohorts, for example athletes with high training volumes or individuals returning from injury, for whom high‐workload exercise modalities are not feasible, or when there is inadequate time available to complete a requisite pre‐intervention exercise test (e.g., VO_2max_ test).

### 4.2 Sustained Attention During Exercise in the Heat Is Improved With Heat Acclimation

Sustained attention in post HST_CMDT_ improved similarly in both groups (+10 ± 4%), consistent with the comparable thermoregulatory adaptations achieved. This improvement exceeded the variation observed during a separate reliability and learning effects experiment in our laboratory (see Data S1). We initially hypothesized that BFR_HA_ would yield smaller improvements in sustained attention due to reduced external workload relative to CTRL_HA_; however, both protocols induced similar thermophysiological adaptation, which aligns with the equivalent enhancement of CMDT performance observed. This finding agrees with the only study examining the effects of HA on time perception during walking in the heat [47] and supports the notion that reduced internal strain through HA, resulting in a less physiologically stressful experience, may help preserve cognitive performance during exercise in the heat. Indeed, in temperate conditions, previous work has similarly associated lower internal strain, for example during low‐intensity cycling, with preserved cognitive performance relative to higher‐intensity exercise [42]. To test the theoretical relationship between core temperature and cognitive performance, whereby reduced hyperthermia is thought to preserve cognition [43], we performed correlational analyses between the changes in peak T_rec_ and SART score during CMDT in the heat from pre to post HST_CMDT_. In both groups, the decrease in T_rec_ did not predict improvements in sustained attention, indicating that CMDT performance cannot be explained solely by thermoregulatory factors. Instead, CMDT performance appears to be influenced by multiple physiological and psychological contributors [4].

Cognitive tasks elicit activation across several cerebral networks depending on task demands [48]. When a physical exercise task is superimposed, overlapping activation occurs in regions such as the prefrontal cortex, leading to competition for shared neural resources (e.g., cerebral blood flow, glucose availability, neurotransmitter turnover) [4]. This competition may partly explain the decline in cognitive performance often observed under situations increasing significantly the internal strain (e.g., high‐intensity exercise, heat). Hyperthermia induced by heat stress (i.e., a 1.2°C –1.5°C increase in T_rec_ from rest) has been shown to modulate prefrontal activity, reflecting increased recruitment of neural resources compared with temperate conditions [11]. HA may attenuate this neural competition, thereby helping to preserve sustained attention during cycling in the heat. To explore the relationship between the central nervous system's ability to generate VA and improvements in sustained attention from pre to post HST_CMDT_, a secondary correlational analysis was conducted. Interestingly, only the BFR_HA_ group demonstrated preserved VA in post HST_CMDT_ visit under fatigue conditions (cf. IMVC_sust_). This improvement was moderately associated with better SART score (*r* = 0.51) during cycling in the heat. While VA provides limited specificity regarding spinal versus supraspinal contributions, this observation warrants further investigation using approaches that more directly assess cortical function (e.g., neuroimaging or transcranial magnetic stimulation). Such mechanisms could involve alterations in cortical excitability or inhibition, as reduced cortical inhibition has been linked to enhanced cognitive performance, although the specific cortical regions involved remain unclear [49].

### 4.3 A BFR‐Based Training Program on Fatigue Resistance

Our final hypothesis focused on the specific neuromuscular adaptations induced by cycling with BFR prior to HWI HA. As a reminder, even for the same perceived effort, exercising with BFR would induce greater nociceptive and metabolic perturbations, thereby enhancing group III/IV afferent feedback [18, 19], which could partly explain the reduced VA typically observed with this method [20]. We hypothesized that a BFR‐based training program with intermittent cycling prior to HWI would further mitigate the central fatigue typically induced by demanding CMDT and heat stress [3, 50]. At the peripheral/muscular level, peak twitch amplitude was similarly decreased in pre and post HST_CMDT_ visits, irrespective of the group. For CTRL_HA_, these findings were expected, as previous studies have reported that HA alone does not markedly affect peripheral components of fatigue [9]. Regarding the BFR condition, little is known about adaptations in terms of exercise‐induced peripheral fatigue (as most studies have focused on hypertrophy or strength adaptations), thereby framing the suggestion that a short BFR‐based training program does not further increase resistance to peripheral fatigue, at least when relative perceived intensities are matched. One may argue that the reduced cycling power during the HA sessions in the BFR_HA_ group could have counteracted the potential to tolerate greater metabolic stress in post HST_CMDT_ visits [51, 52].

Turning to the central component of neuromuscular fatigue, as illustrated by VA assessment at the end of IMVC_sust_, we observed that only the BFR_HA_ group mitigated the decline in VA, which aligned with a lower force loss following HA, despite a comparable amount of force produced during the first seconds of IMVC_sust_ in the post HST_CMDT_ visit (⁓602 N vs. ⁓561 N during the pre HST_CMDT_ visit). For CTRL_HA_, this outcome was somewhat unexpected, as previous research has reported a protective effect of HA on the central nervous system, also assessed during a sustained contraction [9]. It is possible that the type and duration of HA influenced these adaptations. Racinais et al. employed a passive HA protocol across eleven sessions (66 min at 50°C and 50% relative humidity), achieving an end‐session T_rec_ of 39.2°C. This approach likely resulting in greater internal strain than our study (i.e., end‐session T_rec_ of 38.8°C during each HA session). Conversely, BFR_HA_ appeared to benefit from these adaptations, supporting two possible interpretations. First, trained individuals may require an additional homeostatic disturbance (as provided by BFR) during a short HA protocol to elicit meaningful neuromuscular central adaptations, particularly when improved fatigue resistance is targeted. Second, repeated exposure to the exacerbated central fatigue induced by BFR during HA sessions may have enhanced motor command drive, thereby underlined by the gain in IMVC_sust_ [53]. Further investigations should explore the effects of BFR‐based training programs on central mechanisms and fatigue resistance, to determine whether the present findings are replicable, ideally using additional neurophysiological techniques such as transcranial magnetic stimulation or H‐reflex measurements, and to do so across participants of different training status.

### 4.4 Strengths and Limitations

The present study builds on the understanding acquired from existing HA interventions while introducing several novel aspects. First, the use of self‐regulated exercise before HWI and as a pacing strategy during heat stress testing provided a simple, practical methodology for sports settings without inducing premature exhaustion. Second, the inclusion of a cognitive task superimposed on cycling allowed assessment of CMDT performance alongside classical thermoregulatory variables after HA. Finally, adding a BFR group offers an applicable approach for individuals who need to acclimate without high external loads (e.g., athletes in a tapering period, injured individuals) or without maximal‐effort prerequisites (e.g., VO_2_max testing).

However, some limitations should be acknowledged. A first limitation concerns the specificity of the participant sample, which comprised primarily trained males. While less‐trained individuals would likely exhibit similar adaptations, it remains uncertain whether females would respond in the same way, given known sex differences in thermoregulation [54, 55]. Furthermore, the absence of a BFR‐only control group (without HWI) must be acknowledged. While we remain confident that the exercise stimulus alone was insufficient to induce significant aerobic adaptations, given the short duration of the intervention and the high fitness level of the participants [29], it is possible that the unique metabolic and cardiovascular strain of high‐intensity interval exercise combined with BFR could, by itself, trigger physiological adaptations that overlap with those typically induced by HA [24]. Another methodological limitation is the 45‐s delay between HST_CMDT_ cessation and the start of IMVC_brief_ assessment, combined with the transition to a temperate testing environment (cf. safety reasons) during pre and post HST_CMDT_ visits. Taken together, these factors may have permitted partial recovery, potentially masking HA‐induced neuromuscular alterations. Indeed, peripheral fatigue was assessed only from the IMVC_brief_ (i.e., no low‐frequency doublet or peak twitch was measured after the IMVC_sust_), which limits interpretation given that changes in neuromuscular function only occurred during the IMVC_sust_ assessment.

## 5 Conclusion

Overall, significant physiological and cognitive adaptations were observed across both interventions, despite the reduced workload with BFR being likely counterbalanced by comparable internal strain. Following the interventions, sustained attention during challenging exercise in the heat was significantly improved, yet this enhancement was not directly associated with reduced hyperthermia, supporting the view that CMDT performance relies on a multifactorial, holistic trainability. Additionally, cycling with BFR prior to HWI was associated with enhanced fatigue resistance and mitigated central fatigue, suggesting complementary adaptations on the neural component. This study provides preliminary evidence that a BFR‐based training program within a HA intervention can modulate both thermoregulatory and neuromuscular resistance.

## 6 Perspective

This heat acclimation protocol provides several new applied insights. By employing a simultaneous cognitive‐motor dual‐task paradigm during intense exercise in the heat, we imposed a highly challenging situation where the risks of cognitive failure, exacerbated fatigue, and hyperthermia are maximized [56]. This scenario, inevitable for elite athletes or soldiers deployed in desert environments, can be effectively managed through a short‐term heat acclimation consisting of 6 days of high‐intensity intervals followed by hot water immersion at 40°C. Notably, using intervals based on perceived effort extends the practicability of this method to populations unable to perform maximal physical tests [15]. Furthermore, the use of intermittent blood flow restriction provides a first framing of its complementary effect with traditional heat acclimation by lowering the fatigue induced by demanding dual‐tasks. This tool is of particular interest for individuals seeking to reduce external mechanical loads while maintaining high internal loads, such as post‐operative patients, the elderly, or athletes in a tapering period [57]. Overall, these findings offer a versatile framework for preserving performance and safety in extreme thermal conditions.

## Author Contributions

All authors were involved in the design of the study. Goepp Thomas and Hayes Mark performed the data collection. Goepp Thomas drafted the first version of the manuscript, with all authors involved in subsequent revisions and approval of the final document.

## Funding

The authors have nothing to report.

## Conflicts of Interest

The authors declare no conflicts of interest.

## Supporting information


**Data S1:** Detailed procedures and results of the reliability and learning effect related to the SART task.

**Data S2:** Cycling power, rectal temperature and heart rate across the six HA sessions.

**Data S3:** Data of the perceived quadriceps muscle pain throughout the test and neuromuscular indices from IMVC_brief_ in both HA groups.

## Data Availability

The data that support the findings of this study are openly available in HABFR folder at https://osf.io/ekvcg/overview?view_only=cb9837feb2fb43e79879a81547f5e364.
