# Supplementary material for: Heat Acclimation with Blood Flow Restriction Improves Cognitive‐Motor Dual‐Task Ability and Neuromuscular Fatigue
Source: Scand J Med Sci Sports. 2026 Apr 30;36:e70282. doi: 10.1111/sms.70282 (PMC13132146; doi:10.1111/sms.70282)
Supplement: Supplementary file 1 — Data S1: Detailed procedures and results of the reliability and learning effect related to the SART task. [file SMS-36-e70282-s001.docx]

**Supplementary Material 1:** Detailed procedures and results of the reliability and learning effect related to the SART task.

**Methods**

An additional group of trained adults (Tier 2) was recruited *a posteriori*, matched on age and training per week with the initial heat acclimation group (n=26, age: 35 ± 11 years, body mass: 72 ± 10, body mass index: 22.8 ± 2.5 kg.m^-2^, training per week: 8 ± 4 h). They completed two experimental sessions in the laboratory, separated by 9 ± 2 days. During each session, participants performed the SART at rest using the same settings as the HA group (i.e., two 7-min blocks, 800-ms inter-stimulus interval, with 11% of trials presenting the number 3). Before the first session, participants were briefly familiarised with the SART using the same procedure as in the familiarisation session. Before the second session, participants completed a short practice block (30 trials, ~30 s) to refresh task mechanics.

The test-retest reliability was assessed for each block using the Intraclass Correlation Coefficient (ICC) [1]. The learning effect was assessed using a two-way repeated measures ANOVA with main effect of *test-retest* (session 1, session 2) *×* *time intra* (block 1, block 2).

**Results**

A moderate reliability for block 1 [ICC_(3,1)_=0.51] and good reliability for block 2 [ICC_(3,1)_=0.78] was observed. No effect of *test-retest* (P=0.11), *time intra* (P=0.57) or *test-rest ×* *time intra* interaction (P=0.21) was observed.

**References**

[1] T. K. Koo and M. Y. Li, “A Guideline of Selecting and Reporting Intraclass Correlation Coefficients for Reliability Research,” *J. Chiropr. Med.*, vol. 15, no. 2, pp. 155–163, Jun. 2016, doi: 10.1016/j.jcm.2016.02.012.
