# Supplementary material for: Heat Acclimation with Blood Flow Restriction Improves Cognitive‐Motor Dual‐Task Ability and Neuromuscular Fatigue
Source: Scand J Med Sci Sports. 2026 Apr 30;36:e70282. doi: 10.1111/sms.70282 (PMC13132146; doi:10.1111/sms.70282)
Supplement: Supplementary file 2 — Data S2: Cycling power, rectal temperature and heart rate across the six HA sessions. [file SMS-36-e70282-s003.docx]

**Supplementary Material 2:** Cycling power, rectal temperature and heart rate across the six HA sessions.

*Self-regulated cycling phase*. For mean cycling power over the four high-intensity sets, a main effect of *HA* (F_(5, 90)_=4, P=0.004, η_p_^2^=0.17) and *group* (F_(1, 18)_=56, P<0.001, η_p_^2^=0.78) was reported (**FIGURE 1A Sup 2**) with no *HA × group* interaction (P=0.70). Self-regulated powers were increased in S6 compared to S1 (+16 ± 10 W, P=0.006) and S2 (+14 ± 10 W, P=0.026), in both groups. For end HR over the last high-intensity set, no effect of *HA* (P=0.80), *group* (P=0.15) or *HA × group* interaction (P=0.80) was reported (**FIGURE 1B Sup 2**). For end T_rec_, no effect of *HA* (P=0.092), *group* (P=0.83) or *HA × group* interaction (P=0.73) was reported (**FIGURE 1C Sup 2**).

*Hot water immersion phase*. For end HR over the last five minutes of HWI, a main effect of *HA* (F_(5, 90)_=9, P<0.001, η_p_^2^=0.33) was reported (**FIGURE 2A Sup 2**) with no *group* effect (P=0.66) or *HA × group* interaction (P=0.14). HR decreased from S4 (-7 ± 5 bpm, P=0.008) to S6 (-10 ± 6 bpm, P<0.001) compared to S1. For end T_rec_, a main effect of *HA* (F_(5, 90)_=3, P=0.009, η_p_^2^=0.15) was reported (**FIGURE 2B Sup 2**) with no *group* effect (P=0.57) or *HA × group* interaction (P=0.79). T_rec_ was reduced in S6 compared to S1 (-0.25 ± 0.06°C, P=0.040).

**FIGURE 1 Sup 2** Mean cycling power over the four high-intensity sets (panel A), heart rate (HR, panel B) and rectal temperature (T_rec_, panel C) over the last high-intensity set during the cycling phase of each heat acclimation session (from S1 to S6) in the control group (CTRL_HA_), and in the group with blood flow restriction during high-intensity intervals (BFR_HA_). A boxed text presents the *group* effect and P-values for main effects of *heat acclimation* (HA), *group* (G) and *heat acclimation × group* interaction (HA*×*G) are displayed above the figures. Post-hoc statistically significant differences observed for *HA* effect is depicted with the following symbols: * vs. S1 and S2: P-value ≤0.026.**FIGURE 2 Sup 2** Heart rate (HR, panel A) and rectal temperature (T_rec_, panel B) over the last five minutes of the hot water immersion (HWI) phase of each heat acclimation session (from S1 to S6) in the control group (CTRL_HA_), and in the group with blood flow restriction during high-intensity intervals (BFR_HA_). A boxed text presents the *group* effect and P-values for main effects of *heat acclimation* (HA), *group* (G) and *heat acclimation × group* interaction (HA*×*G) are displayed above the figures. Post-hoc statistically significant differences observed for *HA* effect is depicted with the following symbols: *(*,*) vs. S1: P-value =0.040 (≤0.008, <0.001).
