# Supplementary material for: Heat Acclimation with Blood Flow Restriction Improves Cognitive‐Motor Dual‐Task Ability and Neuromuscular Fatigue
Source: Scand J Med Sci Sports. 2026 Apr 30;36:e70282. doi: 10.1111/sms.70282 (PMC13132146; doi:10.1111/sms.70282)
Supplement: Supplementary file 3 — Data S3: Data of the perceived quadriceps muscle pain throughout the test and neuromuscular indices from IMVCbrief in both HA groups. [file SMS-36-e70282-s002.docx]

**Supplementary Material 3:** Data of the perceived quadriceps muscle pain throughout the test and neuromuscular indices from IMVC_brief_ in both HA groups.

*Perceived quadriceps muscle pain*. A main effect of *HA* (F_(1, 18)_=13, P=0.002, η_p_^2^=0.41) was reported (**FIGURE 1A/B Sup 3**) with no *group* effect (P=0.80) or *HA × group* interaction (P=0.77). Quadriceps muscle pain was reduced in post HST_CMDT_ visit (-12 ± 7 a.u., P=0.002) for both groups.

*Neuromuscular indices from IMVC_brief_ (VA and low-frequency to high-frequency ratio).* For the change in VA and low-frequency to high-frequency ratio, no main effects of *HA* (P=0.31 and P=0.21), *group* (P=0.12 and P=0.61, respectively) or *HA × group interaction* (P=0.88 and P=0.65, respectively) were observed (**FIGURE 2A/B Sup 3**).

**FIGURE 1 Sup 3** Perceived quadriceps muscle pain deltas from pre HST_CMDT_ to post HST_CMDT_ (panel A) and raw data at Ex21 (panel B) in the control group (CTRL_HA_), and in the group with blood flow restriction during high-intensity intervals (BFR_HA_). For example, a negative value of Δquadriceps muscle pain at Ex21 in panel A indicates that perceived quadriceps muscle pain is reduced compared to pre HST_CMDT_ visit at this time-point. A boxed text presents the *group* effect (panel A) and P-values for main effects of *heat acclimation* (HA), *group* (G) and *heat acclimation × group* interaction (T*×*G) are displayed above the panels. Post-hoc statistically significant differences observed for *HA* effect is depicted with the following symbol: **: P-value ≤0.006.

**FIGURE 2 Sup 3** Voluntary activation (VA, panel A) and low-frequency to high-frequency ratio (LF/HF, panel B) changes during the brief IMVC (IMVC_brief_), in pre and post HST_CMDT_ visits in the control group (CTRL_HA_), and in the group with blood flow restriction during high-intensity intervals (BFR_HA_). A boxed text presents the *group* effect and P-values for main effect of *heat acclimation* (HA), *group* (G) and *heat acclimation × group* interaction (HA*×*G) are displayed above the figures.
